# An Electrothermal Cu/W Bimorph Tip-Tilt-Piston MEMS Mirror with High Reliability

**DOI:** 10.3390/mi10050323

**Published:** 2019-05-14

**Authors:** Liang Zhou, Xiaoyang Zhang, Huikai Xie

**Affiliations:** Department of Electrical and Computer Engineering, University of Florida, Gainesville, FL 32611, USA; l.zhou@ufl.edu (L.Z.); xzhang292@gmail.com (X.Z.)

**Keywords:** MEMS mirror, electrothermal bimorph, Cu/W bimorph, electrothermal actuation, reliability

## Abstract

This paper presents the design, fabrication, and characterization of an electrothermal MEMS mirror with large tip, tilt and piston scan. This MEMS mirror is based on electrothermal bimorph actuation with Cu and W thin-film layers forming the bimorphs. The MEMS mirror is fabricated via a combination of surface and bulk micromachining. The piston displacement and tip-tilt optical angle of the mirror plate of the fabricated MEMS mirror are around 114 μm and ±8°, respectively at only 2.35 V. The measured response time is 7.3 ms. The piston and tip-tilt resonant frequencies are measured to be 1.5 kHz and 2.7 kHz, respectively. The MEMS mirror survived 220 billion scanning cycles with little change of its scanning characteristics, indicating that the MEMS mirror is stable and reliable.

## 1. Introduction

Microelectromechanical (MEMS) mirrors can actively steer light beams. They play an important role in various optical systems and have been widely used in displays [1,2,3], optical switching [4,5,6], Fourier transform spectroscopy [7,8], optical endomicroscopy [9,10,11,12,13,14], tunable lasers [15,16], structured illumination [17], and light detection and ranging (LiDAR) [18,19]. The development of MEMS mirrors dates back to 1980 when Dr. Kurt Petersen published a seminal paper on a torsional mirror using silicon as the mechanical material [20]. Later, in 1987, Dr. Larry Hornbeck at Texas Instruments successfully invented and developed digital micromirror devices that now dominate the projector market [21]. The market size of MEMS mirrors has been growing for decades, and various MEMS mirrors with advanced features for specific applications are still being developed.

Electrostatic, piezoelectric, electromagnetic, and electrothermal actuations have been commonly used in MEMS mirrors [2]. Every actuation mechanism has its advantages and disadvantages. For instance, electrostatic mirrors usually have the advantages of fast response and low power consumption but at the cost of high driving voltage [2]. Due to the large area of comb drives, the fill factor of the active mirror surface is typically low unless a dedicated mirror transfer process is employed [22]. On the other hand, electrothermal MEMS mirrors have large scan angle, low driving voltage, and high fill factor [11,23,24,25,26], making them especially suitable for biomedical endoscopic imaging applications.

**Figure 1:**
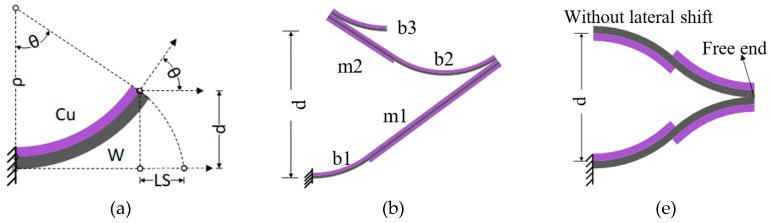

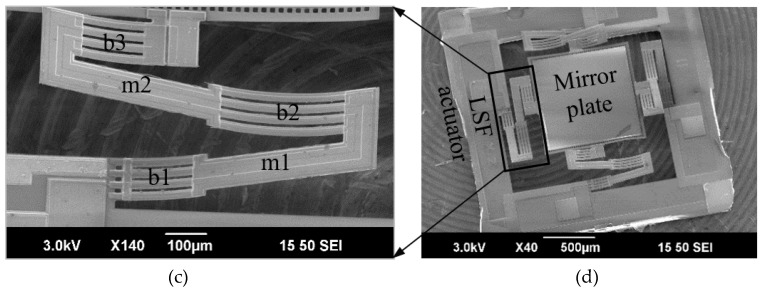
Various bimorph structures: (**a**) A single cantilever bimorph. (**b**) A lateral-shift-free (LSF) bimorph actuator; (**c**) A scanning electron micrograph (SEM) of a Cu/W LSF bimorph actuator; (**d**) An SEM of a Cu/W mirror with LSF design; (**e**) An inverted-series-connected (ISC) bimorph actuator. b1, b2, b3: bimorph segment #1, #2, and #3; m1, m2: multimorph segment #1 and #2.

A variety of MEMS mirrors based on electrothermal bimorph actuators have been reported [23,24,25,26]. An electrothermal bimorph comprises two materials with different coefficients of thermal expansion (CTEs), as shown in Figure 1a. If one end of the bimorph is clamped, the other end will curl up or down as the temperature changes. Cr/SiO_2_ [27], NiCr/SU-8 [28], Au/Si [29], Al/W [30], and Al/SiO_2_ [11,23,24,25,26] material pairs heave been used to form bimorph actuators. The Al/SiO_2_ pair is used most often because of their large CTE difference and their wide processing availability in almost any MEMS or integrated circuit (IC) fabrication facilities. However, Al is a metal with low melting point (660 °C), and is susceptible to creep failure [31]. SiO_2_ is a brittle material, which may result in fracture of bimorphs due to fabrication defects and overstress. Thus, the lifetime and reliability of Al/SiO_2_ bimorph based MEMS mirrors may be limited [31].

Therefore, a new material pair for bimorphs is needed to obtain more reliable MEMS mirrors. Cu and W have high Young’s moduli, their CTE difference is relatively large, and their thermal diffusivities are also large. Thus, high stiffness and fast thermal response can be expected from Cu/W bimorphs. Zhang et al. demonstrated a Cu/W bimorph based electrothermal MEMS mirror using a lateral-shift-free (LSF) bimorph design [32]. The LSF bimorph actuator consists of three Cu/W bimorph segments (b1, b2, and b3) and two Cu/W/Cu multimorph segments (m1, m2), as shown in the Figure 1b,c. By properly choosing the length ratios of these five segments, the LSF bimorph design minimizes the lateral shift of the central mirror plate. This LSF design also achieves large vertical displacement by utilizing temperature-insensitive Cu/W/Cu multimorphs to amplify the displacement generated from the curling Cu/W bimorphs. An SEM of the LSF Cu/W MEMS mirror is shown in Figure 1d; a large piston displacement of 320 μm and a large scan angle of ±18° were obtained [32]. However, due to the long actuator beams, the stiffness of the bimorph actuators is low (only about 0.1 N/m for the design in Figure 1d) and the thermal resistance is large, causing long thermal response time (the thermal time constant was about 6 ms for the design in Figure 1d). In addition, this LSF MEMS mirror’s effective fill factor, that is, the ratio of the area of the mirror plate to the area occupied by both the actuators and mirror plate, is only about 35%. Furthermore, the mirror plate has a small in-plane rotation upon piston actuation because the four actuators are not completely symmetric.

In this paper, we present a new electrothermal Cu/W bimorph MEMS mirror with an inverted-series-connected (ISC) structure. As shown in Figure 1e, an ISC structure achieves vertical displacement through connecting four segments of bimorphs with flipped layers in series. The ISC actuator design was firstly developed by Todd et al. to overcome the lateral shift and the tip-tilt angle of a single bimorph [25]. Compared to the LSF actuator in Figure 1b, this ISC actuator eliminates the long and wide multimorphs that deteriorates the fill factor and resonant frequency. Thus, this ISC actuator design can increase both stiffness and fill factor. At the same time, this ISC bimorph actuator design can be made completely symmetric, in which every bimorph is the same except the layer sequence. The W layer of the bimorphs also functions as heaters. This concept was initially reported in [33], where a downward ISC Cu/W mirror was reported with preliminary results. This paper focuses on the design, optimization, fabrication, and characterization of an upward ISC Cu/W mirror.

In the following, the bimorph material selection process is discussed in Section 2, device design including structure parameters and simulation is presented in Section 3, the detailed device fabrication process is introduced in Section 4, and the device characterization including quasi-static, dynamic, and long-term stability tests is presented in Section 5.

## 2. Material Selection

Material selection is crucial to designing reliable bimorphs for MEMS mirrors. Thin film dielectric materials are fragile, so metals are preferred. For metal microstructures, creep and fatigue are among the most important concerns [31]. Alloys are often used over pure metals. For example, Al alloys are successfully used by Texas Instruments to reduce the creep of digital micromirror devices [31]. However, alloys with proper compositions are often difficult to find especially for MEMS processes-compatible ones, so only the pure metals commonly used in MEMS or semiconductor industries, as listed in Table 1, were considered. Evaluation of the stiffness, bimorph responsivity, response time, and maximum working temperature of a single bimorph with two materials of the same width was used to select the two materials.

The tilt angle at the end of the bimorph was determined by the intrinsic stresses and the extrinsic stresses in the two thin-film layers. The intrinsic stresses, which are incurred by the materials and deposition temperature, determined the initial tip-tilt angle and displacement. Miniaturized intrinsic stresses were expected to make the mirror surface at the same level as the substrate, facilitating the fabrication and applications. W was just the right material whose residual stress could be well controlled through adjusting the argon pressure or substrate temperature during sputtering.

The bimorph responsivity, defined as the ratio of the rotation angle at the end of the bimorph, Δθ, over the temperature change, ΔT, is expressed as [35]:

(1) $$\Delta \theta /\Delta T=\frac{\beta_{b}l}{t_{a}+t_{b}}\left(\alpha_{a}-\alpha_{b}\right),$$

where $\alpha_{a}$ and $\alpha_{b}$ are the CTE’s of material a and b, respectively, $t_{a}$ and $t_{b}$ are the thicknesses of material a and b, respectively, $\beta_{b}$ is the curvature coefficient of the bimorph, and l is the length of the bimorph. According to Equation (1), the bimorph responsivity is proportional to the CTE difference. Thus, Al and SiO_2_ are often chosen as the bimorph materials because of their large CTE difference of 23.2 × 10^−6^/K. The CTE difference between Al and W [30] is comparable to that of Al and SiO_2_, but Al would incur creep. Although the CTE difference between Cu and W is only around 60% of that of Al and SiO_2_, their melting points are much larger than that of Al. Therefore, Cu and W bimorphs can work at higher temperature to achieve similar rotation angles as Al and SiO_2_ bimorphs. In addition, creep is smaller for a metal with higher melting temperature, so the creep failure of Cu/W MEMS mirrors will be greatly reduced. Note that the temperature change for a given electrical power is determined by the thermal resistance between the bimorph and the substrate as well as the heat loss to the air via convection; more details can be found in [36].

The equivalent stiffness of the bimorph in Figure 1a can be found as:

(2) $$k=\frac{3EI}{l^{3}},\;\mathrm{where}\;EI=\frac{wt_{b}^{3}t_{a}E_{b}E_{a}}{12\left(t_{a}E_{a}+t_{b}E_{b}\right)}K_{1},\;\mathrm{and}\;K_{1}=4+6\frac{t_{a}}{t_{b}}+4{\left(\frac{t_{a}}{t_{b}}\right)}^{2}+\frac{E_{a}}{E_{b}}{\left(\frac{t_{a}}{t_{b}}\right)}^{3}+\frac{E_{b}}{E_{a}}\frac{t_{b}}{t_{a}}.$$

Therefore, the stiffness is highly dependent on the Young’s moduli of the materials a and b as well as their thickness ratio. The equivalent rigidity of a Cu/W bimorph with a same width and equivalent thickness is around three times of that of an Al/SiO_2_ bimorph. In other words, compared to an Al/SiO_2_ bimorph, a Cu/W bimorph with a much smaller thickness can be used to achieve the same stiffness.

From a simplified one-dimensional thermal lumped model, the thermal response time is inversely proportional to the thermal diffusivity (*α*), that is,

(3) $$t\propto R_{\mathrm{th}}C_{\mathrm{th}}\propto \frac{1}{k/\rho c_{p}}=\frac{1}{\alpha },$$

in which thermal convection and radiation are neglected for simplification. The thermal diffusivities of Cu and W are comparable to Al (120% and 70% of that of Al, respectively), but over 75 times higher than that of SiO_2_. Therefore, the thermal response time of a Cu/W bimorph will be much smaller than that of an Al/SiO_2_ bimorph.

For a reliable bimorph, the materials must work in the region of elasticity, that is, the maximum bending stress must not exceed the yield strength. According to Table 1, the yield strengths of Cu and W are about two times and four times higher than that of Al, respectively. In addition, Cu and W are widely available for micromachining and their fabrication processes are mature. Also, W, whose resistivity is 5.6 × 10^−8^ Ω·m, is commonly used in incandescent light bulbs. Therefore, the W layer can function as a heater.

With all the above merits, Cu and W were selected as the materials for making the ISC bimorph actuators in this work.

## 3. Device Design

**Figure 2:**
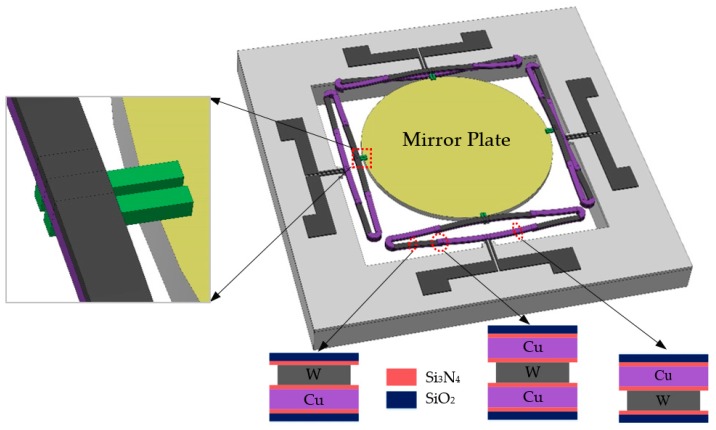
Schematic of a microelectromechanical (MEMS) mirror based on Cu/W ISC actuators.

The schematic of the MEMS mirror built on the Cu/W bimorphs with ISC structures is shown in Figure 2. The central mirror plate was made of a 20-µm-thick silicon for optical flatness and a 0.2-µm-thick aluminum on the surface for high reflectance. The mirror plate was 1 mm in diameter and suspended by four pairs of ISC actuators. There were thin silicon oxide beams between the ISC actuators and the mirror plate, as shown in the inset of Figure 2, functioning as thermal isolation to confine the Joule heat to the bimorphs and minimize the temperature rise on the mirror plate. There was another set of silicon oxide beams between the bimorph actuators and the substrate, forming a thermal barrier to reduce the heat to the silicon substrate. There were eight pads extended from the tungsten layer of the bimorphs with two pads on each side of the substrate. Thus, every actuator could be actuated separately. The mirror plate could move vertically when all the actuators were applied with the same voltage, and could rotate when the four actuators were applied with different voltages.

If the W and Cu layers have the same width, the optimal thickness ratio of these two layers is 0.56 for achieving the maximum displacement [23]. However, the actual widths of the Cu and W layers were different. For the sake of good step coverage and reliable photolithography, the Cu layers were chosen to be wider than the W layers. In this design, the width of the Cu layer was set as 30 µm, while the W width was set as 16 µm, which ensured the W layer was either fully covered by the Cu layer or fully on top of the Cu layer, even with minor mask aligning errors. The structure parameters of the Cu/W ISC MEMS mirror are given in Table 2. With the aid of COMSOL simulation, it was found that the optimal W-to-Cu thickness ratio is 0.77. By considering the required robustness of the MEMS mirror and easy fabrication, the actual W and Cu thicknesses were chosen as 1.0 μm and 1.3 μm, respectively. The flexural rigidity (EI) of the Cu/W bimorph was 4.82 $\mathrm{Pa}\cdot {\mathrm{mm}}^{4}$, which is 4.25 times that of the Al/SiO_2_ bimorph in [3] whose Al and SiO_2_ thicknesses were 1.1 μm and 1.2 μm, respectively.

**Figure 3:**
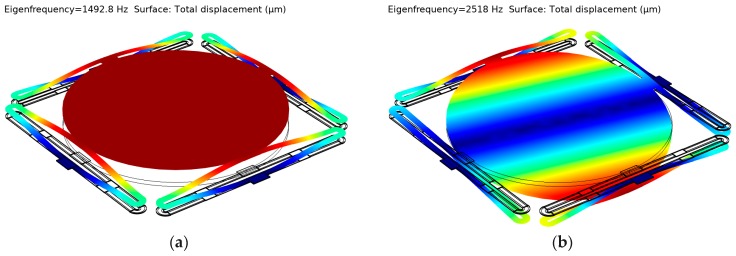
The modal simulation of the Cu/W mirror. (**a**) First resonant mode, piston, at the frequency of 1.493 kHz; (**b**) Second resonant mode, tip-tilt, at the frequency of 2.518 kHz.

Note that even when Cu is passivated with SiO_2_, oxygen can still diffuse through and oxidize Cu, so a thin layer (~50 nm) of Si_3_N_4_ is needed as a diffusion barrier layer to wrap Cu layers. A finite element 3D model with the parameters as shown in Table 2 was created in COMSOL Multiphysics (version 5.4, COMSOL Inc., Stockholm, Sweden) to show the performance of the MEMS mirror. All layers including the dielectric layers were considered. As shown in Figure 3, the first and second resonant frequencies were 1.493 kHz and 2.518 kHz, respectively. Since the mass of the mirror plate was several orders of magnitude larger than those of the actuators, the stiffness of a single actuator can be calculated by:

(4) $$k_{\mathrm{act}}=\frac{1}{4}m_{\mathrm{plate}}{\left(2\pi f_{p}\right)}^{2},$$

where *m*_plate_ is the mass of the mirror plate, and *f_p_* is the piston resonant frequency. Thus, the stiffness of one double S-shaped bimorph actuator is $k_{\mathrm{act}}=0.81\;N/m$. Tip-tilt actuation can be realized by applying different temperature at the four different actuators. Raising the same temperature on four actuators at the same time results in a piston movement.

## 4. Device Fabrication

**Figure 4:**
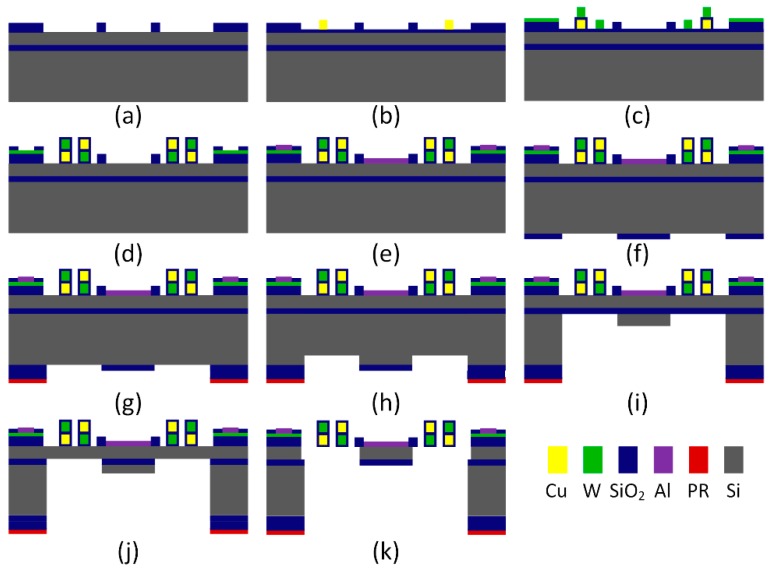
Fabrication process flow of the MEMS mirror.

The mirror pate and the bimorphs were released by bulk micromachining. The fabrication process flow is illustrated in Figure 4. First, a 1-μm-thick plasma enhanced chemical vapor deposition (PECVD) SiO_2_ was deposited on a 4” silicon on insulator (SOI) wafer and wet etched to form electrical insulation on top of the silicon device layer and thermal isolation from the bimorphs to the substrate and to the mirror plate (Figure 4a). A 0.15/0.05-μm PECVD SiO_2_/Si_3_N_4_ was deposited and reactive-ion-etch (RIE) patterned as the bottom diffusion barrier layer of the bimorphs. A 1.3 μm Cu layer was sputtered and lift-off to define the bimorphs that require Cu as the bottom layer (Figure 4b). A 0.1 μm Si_3_N_4_ layer was deposited and RIE patterned for electrical isolation and a 1 μm W layer was sputtered and patterned via lift-off to define the bimorphs; the W layer also worked as the resistor for Joule heating (Figure 4c). Another 0.1 μm Si_3_N_4_ layer was deposited on top of the W layer and vias were opened on top of W by RIE. The second Cu layer was then sputtered and lift-off to define the bimorphs that required Cu as the top layer, followed by another 0.05/0.15 μm thin PECVD Si_3_N_4_/SiO_2_ deposited as the diffusion barrier layer of the bimorphs (Figure 4d). These Si_3_N_4_/SiO_2_ multilayer dielectric layers between the bimorphs were etched by RIE for later release, and vias were formed on top of the Cu layer. A 0.5 μm Al layer was sputtered to define the mirror surface and the bonding pads on top of Cu (Figure 4e). At this point, all the processes on the front side of the wafer were done.

After the wafer was flipped over, a 0.2 μm SiO_2_ was deposited and RIE etched to define the regions corresponding to the bimorphs (Figure 4f). Next, a photoresist pattern corresponding to the entire bimorphs plus the mirror plate was formed (Figure 4g). Then, a first round of deep reactive-ion-etching (DRIE) was used to etch trenches into the silicon substrate by about 40 μm while the silicon under the mirror plate was still intact (Figure 4h). Next, the 0.2 μm SiO_2_ was removed by RIE. A second round of DRIE was used to etch down to the buried oxide (BOX) layer (Figure 4i), followed by removing the BOX layer with RIE (Figure 4j). Finally, a third round of DRIE was done to remove all the remaining silicon layer under the bimorphs and the mirror plate (Figure 4k). As the front-side Al mirror surface was not exposed to DRIE, the surface quality of the mirror was high.

**Figure 5:**
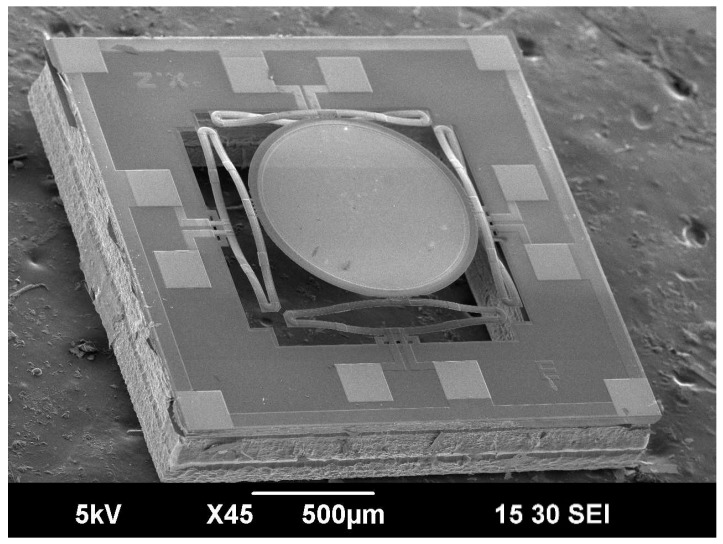
SEM of a fabricated MEMS mirror.

An SEM of a fabricated ISC MEMS mirror is shown in Figure 5. The device footprint was 2.2 mm × 2.2 mm with an effective fill factor of 48% even with a circular mirror plate, which was still 37% larger than the LSF design in Figure 1d. The initial elevation of the mirror plate was measured to be 128 μm, incurred by the residual stresses, and the bimorphs were free of oxidation. The measured resistances of the four actuators were 30.4–32.4 Ω at room temperature.

## 5. Characterization

The quasi-static, dynamic and frequency responses of the MEMS mirror were characterized. The long-term reliability was also tested. These experimental results are presented below.

### 5.1. Static Response

**Figure 6:**
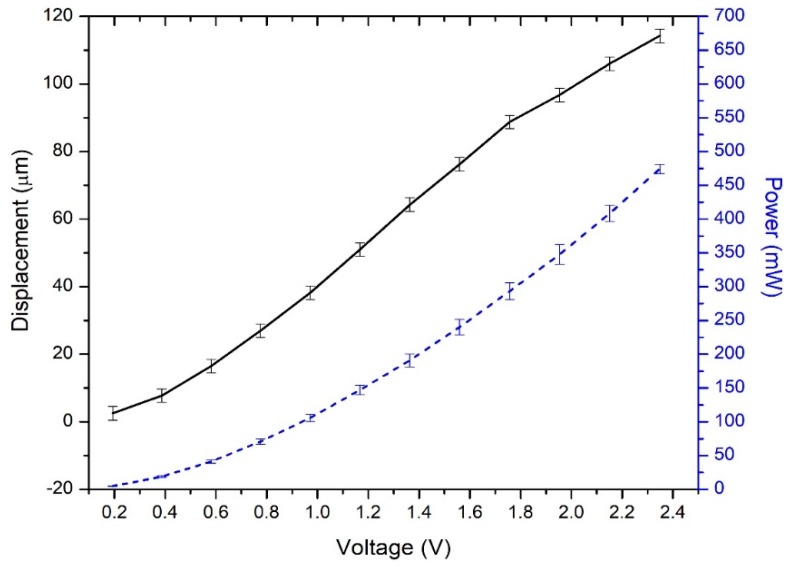
The vertical displacement (solid line), and the corresponding consumed power (dash line) versus the applied voltage. The errors for the displacement measurement were about ± 2 µm resulting from the errors of the microstage position reading and the focal point determination of the optical microscope.

**Figure 7:**
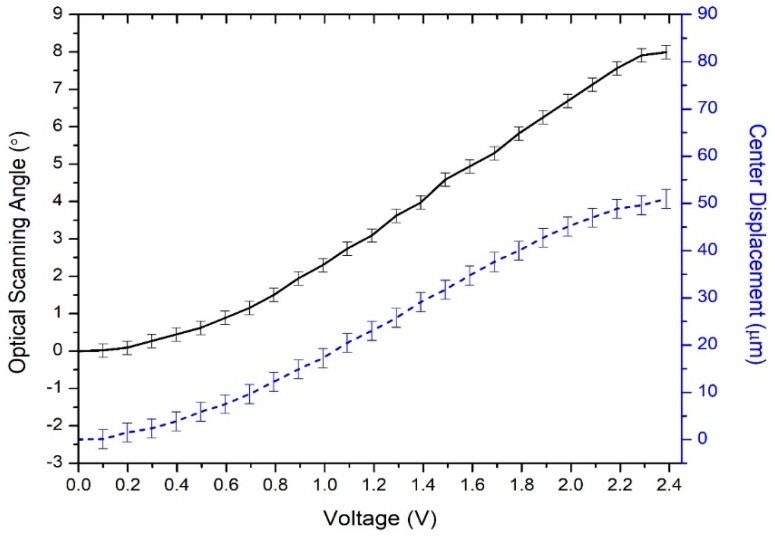
The optical scan angle (solid line), and the corresponding center displacement (dash line) versus the applied voltage.

When a same direct current (DC) voltage was applied to all four actuators, the mirror plate moved vertically. An optical microscope was used to measure the heights of the mirror plate at different DC voltages. Figure 6 plots the piston displacements of the mirror plate versus the applied DC voltage and the corresponding power, respectively, showing that the mirror plate traveled 114 μm at only 2.35 V or 475 mW. When a voltage was applied on one actuator while leaving other three actuators open-circuit, the mirror plate tilted. Figure 7 shows the optical scan angle of the mirror plate versus the applied DC voltage, showing that the mirror plate tilted 4° (or 8° optical angle) at 2.35 V. Also plotted on Figure 7 is the displacement of the mirror plate center reaching to 51 μm at 2.35 V. This means that the mirror plate was flipping instead of rotating along its central axis. In order to keep the center stationary, a differential drive—that is, applying a pair of differential voltages on one pair of opposing actuators with a DC bias set on all four actuators—can be used [23].

### 5.2. Frequency Response

**Figure 8:**
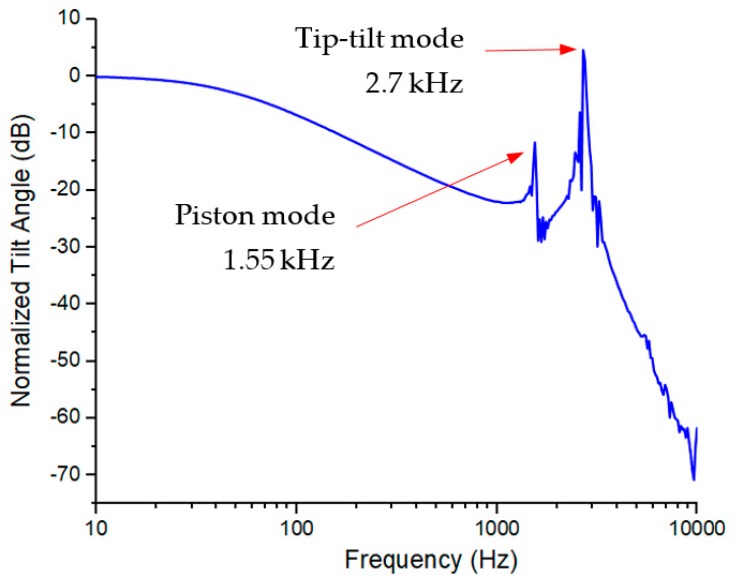
The frequency response of the micromirror from 1 Hz to 10 kHz.

The frequency response was measured by using a network analyzer. An input sweep-frequency voltage signal, 1 + 0.1 × cos(2*πft*) V, generated by the network analyzer, was applied to the MEMS mirror; the laser spot reflected by the scanning mirror plate was picked up by a photosensitive detector (PSD) whose output signal was a measure of the tilt angle of the mirror plate. This signal was sent back to the network analyzer, so the frequency response was directly obtained. The measured frequency response is shown in Figure 8, where the first mode (piston) was 1.55 kHz and the second mode (tip-tilt) was 2.7 kHz. The resonant modes were well predicted by the simulation results (see Figure 3) with an error less than 7%. The Q factor of the tip-tilt mode was 25.5. The 3 dB cutoff frequency, *f*_3dB_, was around 50 Hz, which was the result of the thermal response. Thus the thermal time constant $\tau_{T}=\frac{1}{2\pi f_{3\mathrm{dB}}}=3.2$ ms, which is around 50% of the time of the LSF actuator as shown in Figure 1d [32].

Note that the measured piston resonant frequency was *f_p_* = 1550 Hz, so $k_{\mathrm{act}}=\frac{1}{4}m_{\mathrm{plate}}{\left(2\pi f_{p}\right)}^{2}=0.87\;N/m$, which is in a good agreement with the simulated stiffness and seven times as much as that of the design in Figure 1d. According to Figure 8, the measured displacement was 114 μm at 2.35 V; thus, the force generated by each electrothermal bimorph actuator was about 92.3 μN at that voltage. Therefore, this type of electrothermal actuators is also suitable for applications that require relatively large driving force, such as MEMS lens scanners.

### 5.3. Step Response

**Figure 9:**
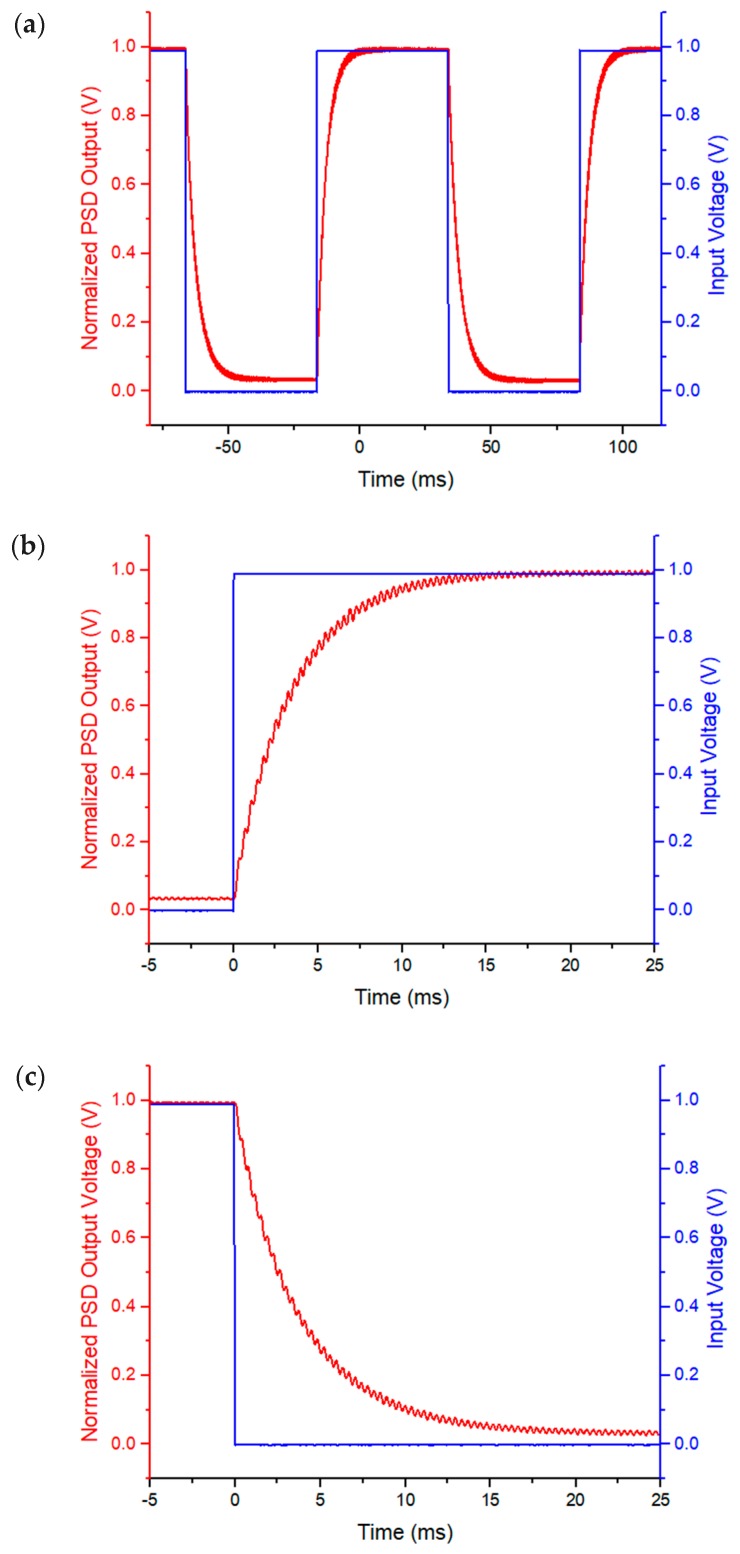
(**a**) Step response of one actuator of the MEMS Mirror; (**b**) zoom-in rise edge; (**c**) zoom-in fall edge.

Step response and frequency response have been characterized. The step response was measured with a laser shinning on the mirror plate and a PSD detecting the position of the reflected laser beam. A 10 Hz square wave with a 50% duty cycle and a 1V amplitude was used to drive a single actuator. The result is shown in Figure 9a, with the zoom-in views of the rise and fall response in Figure 9b,c, respectively. The 10–90% rise and fall time were 7.3 ms and 8.5 ms, respectively. The fall time was 1.2 ms or 16% longer that the rise time, which is believed to be caused by the heat stored inside the mirror plate flowing back into the actuators during cooling. Note that in Figure 9 the step response was smooth with no overshoot and very small ringing. This was the result of the low-pass filtering effect of the thermal response. There is a 20 dB/Decade roll-off after the thermal cut-off frequency. Thus, the resonant peak height was proportional to the thermal response time and inversely proportional to the resonant frequency. According to the finding reported in [37], the overshoot and ringing of a step response of an electrothermal actuator will be greatly suppressed if $\tau_{T}\cdot f_{0}>Q/\left(2\pi \right)$. In this case, $\tau_{T}\cdot f_{\mathrm{tip}-\mathrm{tilt}}=3.2\times 2.7=8.6$, which is greater than *Q*/2*π* = 4.

### 5.4. Reliability

**Figure 10:**
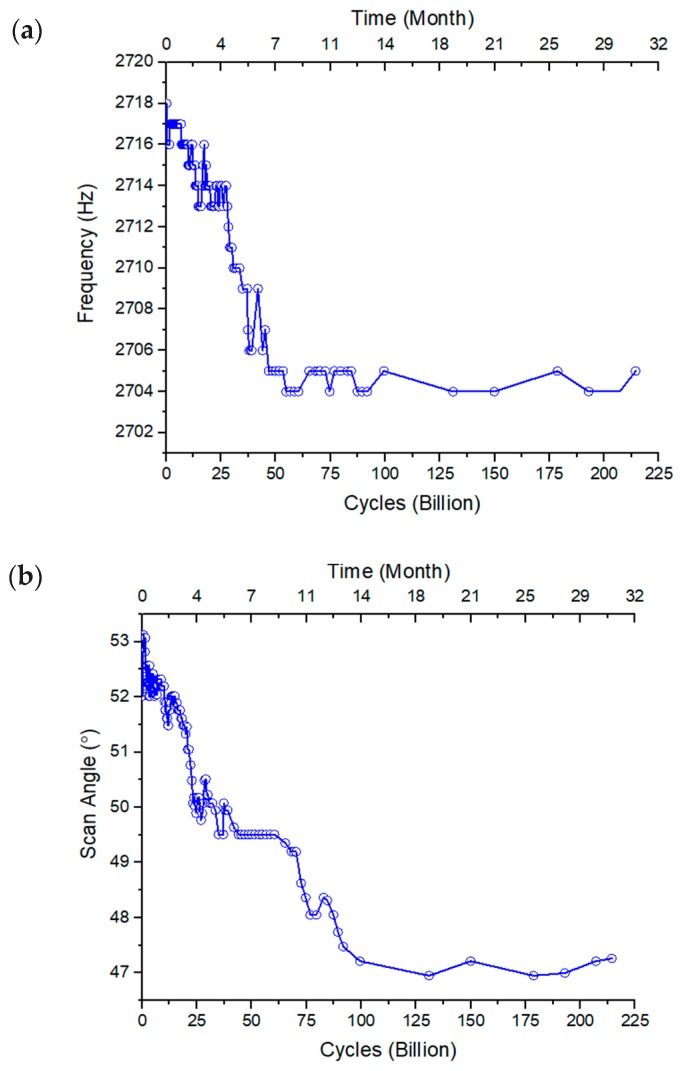
(**a**) Long-term frequency shift; (**b**) long-term scan angle change at the corresponding tip-tilt resonant frequency.

The reliability was characterized by recording the tip-tilt resonant frequency and its corresponding scan angle for more than 30 months. A sine waveform voltage signal with an amplitude of 1 V and an offset of 1 V was applied to one actuator of the fabricated Cu/W ISC MEMS mirror. A laser beam was shined on the mirror plate, and the reflected laser beam was projected to a screen. The driving frequency was continuously adjusted to its resonant frequency to keep the scan angle at its maxima, and both the frequency and the corresponding scan angle were recorded. The resonant frequency and the resonant scan angle over time are plotted in Figure 10. During the first 9 months, the resonant frequency dropped slowly from 2718 Hz to 2705 Hz, corresponding to a frequency shift as small as 0.47%. The resonant scan angle decreased gradually during the first 13 months, dropping about 10.7% (from 53.2° to 47.5°). After 13 months’ continuous running, both the resonant frequency and the scan angle became very stable, maintaining at 2705 ± 1 Hz and 47 ± 0.4°, respectively. Thus, this MEMS mirror is reliable and can be used for long-term operation and even for open-loop driving. Note that even though the overall changes of the resonant frequency and maximum scan angle were small, there still existed a rapid-changing time period, which happened to be the beginning part of the device operation life time. This rapid-changing period is believed to be undergoing the burn-in process. More experiments are needed to verify this.

**Figure 11:**
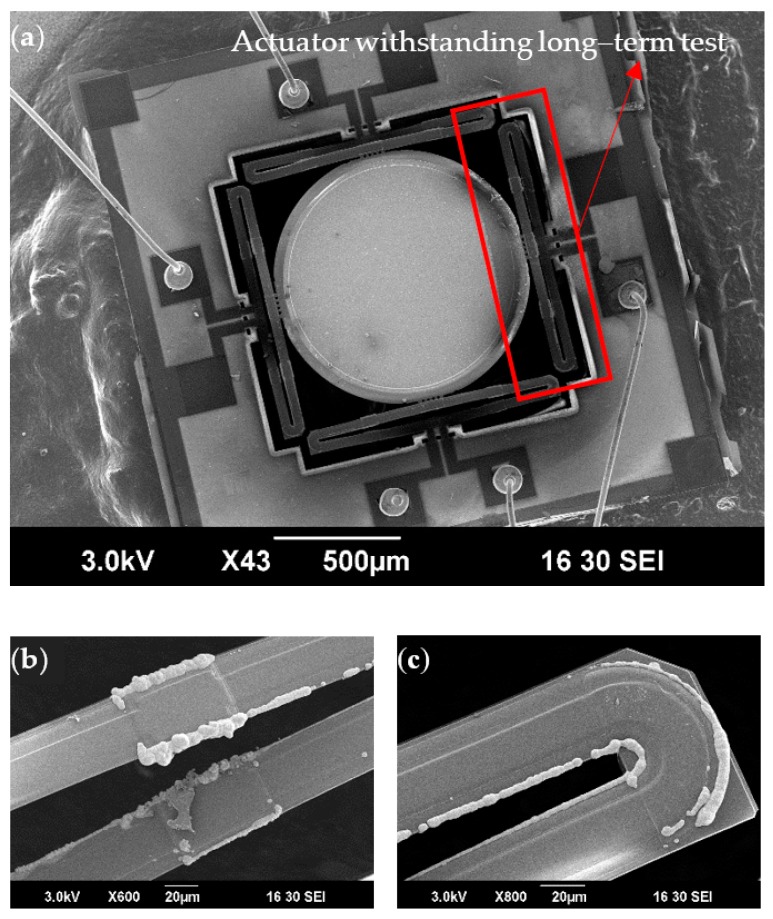
SEM pictures of the MEMS mirror after long-term actuation under the tip-tilt resonant frequency. (**a**) The full device; (**b**) a zoom-in SEM picture of the actuated bimorph near overlap between inversed bimorphs; (**c**) a zoom-in SEM picture of the actuated bimorph near a corner.

Figure 11a shows a scanning electron microscope (SEM) image of the MEMS mirror after the long-term (30 months) test. The entire device still looked clean except the joint part between the mirror plate and the actuated bimorph actuator was contaminated. As shown in the close-view images of the actuated bimorphs (Figure 11b,c), some extrusion accumulated at the edge of the bimorphs. This is believed to be copper oxide. Since the Si_3_N_4_ barrier layer was partially etched during DRIE, some copper atoms diffused out and were oxidized on the surface of the bimorphs. It is also believed that this Cu oxidization accounted for the relatively large changes of the resonant frequency and the scan angle during the initial 13-month long-term test. More study will be performed to understand the observed phenomena.

## 6. Conclusions

A reliable MEMS mirror based on the ISC Cu/W bimorph actuator design has been successfully demonstrated. Compared to its LSF bimorph design counterpart with the same dimensions, this ISC design is completely symmetric with six times higher stiffness, 37% higher fill factor, and 50% smaller thermal response time. In addition, the ISC bimorph MEMS mirror has been proven to be stable long-term, surviving over 200 billion cycles of large angular scanning. In the future, this Cu/W ISC design will be further optimized, including eliminating the Cu oxidation by adopting new diffusion barrier layers, reducing the thicknesses of the insulation layers, minimizing the width difference between the Cu and W layers, and increasing the bimorph width to achieve even larger scan angle, higher robustness, and better reliability.

## Figures and Tables

**Table 1:** Material properties of commonly used MEMS materials [34].

Material	CTE (10^−6^/K)	Thermal Conductivity (W/mK)	Young’s Modulus (GPa)	Melting Point (°C)	Yield Strength (MPa)
Si	3.0	150.0	179	1414	-
SiO_2_	0.4	1.4	70	1700	-
Al	23.6	237.0	70	660	124
Au	14.5	318.0	78	1064	-
Cu	16.9	401.0	120	1083	262
W	4.5	173	410	3410	550
Cr	5.0	93.9	140	1907	200

**Table 2:** Design parameters of Cu/W MEMS Mirror.

Structure Parameters	Value
Device footprint	2.2 mm × 2.2 mm
Diameter of the mirror plate	1 mm
Mirror plate thickness	20 µm
Length of each bimorph	180 µm
Width of W	16 µm
Width of Cu	30 µm
Length of overlap	60 µm
